# Sequence-Based Characterization of Microalgal Microbiomes: Impact of DNA Extraction Protocol on Yield and Community Composition

**DOI:** 10.1128/spectrum.03408-22

**Published:** 2023-03-28

**Authors:** Line Roager, Eva C. Sonnenschein, Lone Gram

**Affiliations:** a Department of Biotechnology and Biomedicine, Technical University of Denmark, Kongens Lyngby, Denmark; University of Minnesota Twin Cities

**Keywords:** DNA extraction, microalgae, microbiome analyses, phycosphere

## Abstract

The bacterial communities associated with microalgae are vital for the growth and health of the host, and engineering algal microbiomes can enhance the fitness of the algae. Characterization of these microbiomes mostly relies on sequencing of DNA, which can be extracted with an array of protocols that potentially impact DNA quantity and quality and thus potentially affect subsequent analyses of microbiome composition. Here, we extracted DNA from Isochrysis galbana, Tetraselmis suecica, and Conticribra weissflogii microbiomes using four different protocols. DNA yield and quality was greatly impacted by the choice of extraction protocol, whereas microbiome composition determined by 16S rRNA gene amplicon sequencing was only impacted to a minor degree, with microalgal host species being the main determinant of microbiome composition. The *I. galbana* microbiome was dominated by the genus *Alteromonas*, whereas the microbiome associated with T. suecica was dominated by *Marinobacteraceae* and *Rhodobacteraceae* family members. While these two families were also prevalent in the microbiome associated with *C. weissflogii*, *Flavobacteriaceae* and *Cryomorphaceae* were also highly dominant. Phenol-chloroform extraction resulted in higher DNA quality and quantity compared to commercial kits; however, because they have other advantages such as high throughput and low toxicity, commercial kits can be employed to great benefit for the characterization of microalgal microbiomes.

**IMPORTANCE** Microalgae are very important as primary producers in the ocean, but also as forthcoming sustainable producers of biotechnologically interesting compounds. Accordingly, the bacterial microbiomes associated with microalgae are attracting increasing attention due to their effects on the growth and health of microalgae. Since most members of these microbiomes cannot be cultured, knowledge about community composition is best obtained using sequencing-based methods. This study evaluates the impact of DNA extraction methods on DNA quantity and quality along with sequence-based characterization of the bacterial microbiome composition of three microalgae: Isochrysis galbana, Tetraselmis suecica, and Conticribra weissflogii.

## INTRODUCTION

Microalgae are the primary producers of the oceans and are responsible for 50% of global CO_2_ fixation. They are also of significant (bio)industrial interest, widely used as feed in aquaculture and as producers of high-value bio-products, including biofuels, in biotechnology (1–3). Understanding and ensuring rapid and stable algal growth is essential in both nature and bio(industrial) production. Algal growth and metabolism are strongly influenced by the microbial community (the microbiome) surrounding the algae (4–7), and axenic (sterile) algae often grow poorly compared to non-axenic algae (8, 9). Also, the production of valuable compounds such as polyunsaturated fatty acids is enhanced by the microbiome (10). The term “phycosphere” describes the region around microalgal cells inhabited by microbes; it is inspired by the term “rhizosphere,” which defines the niche surrounding plant roots in which plants chemically interact with other organisms (11, 12). In the phycosphere, algae release nutrients, including dissolved organic carbon, representing a hot spot for heterotrophic bacteria (13). This competitive microenvironment attracts and harbors diverse microbial taxa with the potential to produce bioactive compounds (7, 14–17).

In many studies, analyses of the algal microbiome have been based on cultivation, isolation, and identification of the microorganisms present (18–20); however, as in most ecological niches, not all microorganisms can be cultured under standard laboratory conditions. Therefore, studies of microbiomes have increasingly relied on culture-independent approaches such as amplicon-sequencing (e.g., 16S rRNA gene) and shotgun metagenomics (21–24). A key first step in any sequence-based analysis is DNA extraction, and a multitude of kits and procedures have been developed for this purpose. Components of the sample can often interfere with particular steps of an extraction protocol (25, 26). Therefore, each sample type requires careful assessment of the DNA extraction protocol with respect to both the amount and quality of DNA extracted, but also to determine whether extraction affects the subsequent microbial profile obtained from sequence-based analysis. For human samples, comparisons of extraction methods have shown varying results, with some studies finding large effects on microbial community composition (27) and others finding small impacts which are overshadowed by other factors, such as the specific host (25, 28). In other sample types, such as marine biofilms, microbial community compositions have been influenced by the DNA extraction methods used (29), whereas the microbial communities of ants were not significantly impacted (26), underlining the need for specific evaluations of the impact of extraction methods on specific sample types.

We and others have studied algal microbiomes relying on phenol-chloroform DNA extraction as a well-known standard practice for amplicon and metagenomics sequencing (15, 16, 24, 30–32). However, it is laborious, time-intensive, and includes hazardous reagents, and using any of the commercially available, standardized kits in a high-throughput format would be preferable. Therefore, the purpose of the present study was to determine how four commonly used DNA extraction protocols affected both the yield and quality of the DNA sample and whether this had any effect on the microbiome composition as determined by 16S rRNA gene amplicon sequencing. We tested these kits on the microbial samples from three algal species—a chlorophyte, a haptophyte, and a diatom—to evaluate whether the methods were suitable across different algal taxa.

## RESULTS AND DISCUSSION

Xenic cultures of the three microalgae grown for 7 days contained 6.1 × 10^6^, 7.1 × 10^5^, and 8.6 × 10^4^ algal cells per mL for Isochrysis galbana, Tetraselmis suecica, and Conticribra weissflogii, respectively. In the attached (A) fraction of bacteria in the microbiomes, *I. galbana* harbored 6.9 × 10^5^ cells/mL, T. suecica harbored 2.3 × 10^5^ cells/mL, and *C. weissflogii* harbored 5.8 × 10^6^ cells/mL. Generally, the free-living (FL) fractions had higher estimates of bacterial cells per mL, with 1.2 × 10^6^ cells/mL in the *I. galbana* culture, 2.3 × 10^6^ in the T. suecica culture, and 3.7 × 10^7^ in the *C. weissflogii* culture. Thus, bacterial loads across the different xenic microalgal cultures were comparable.

### DNA yield and quality.

**FIG 1:**
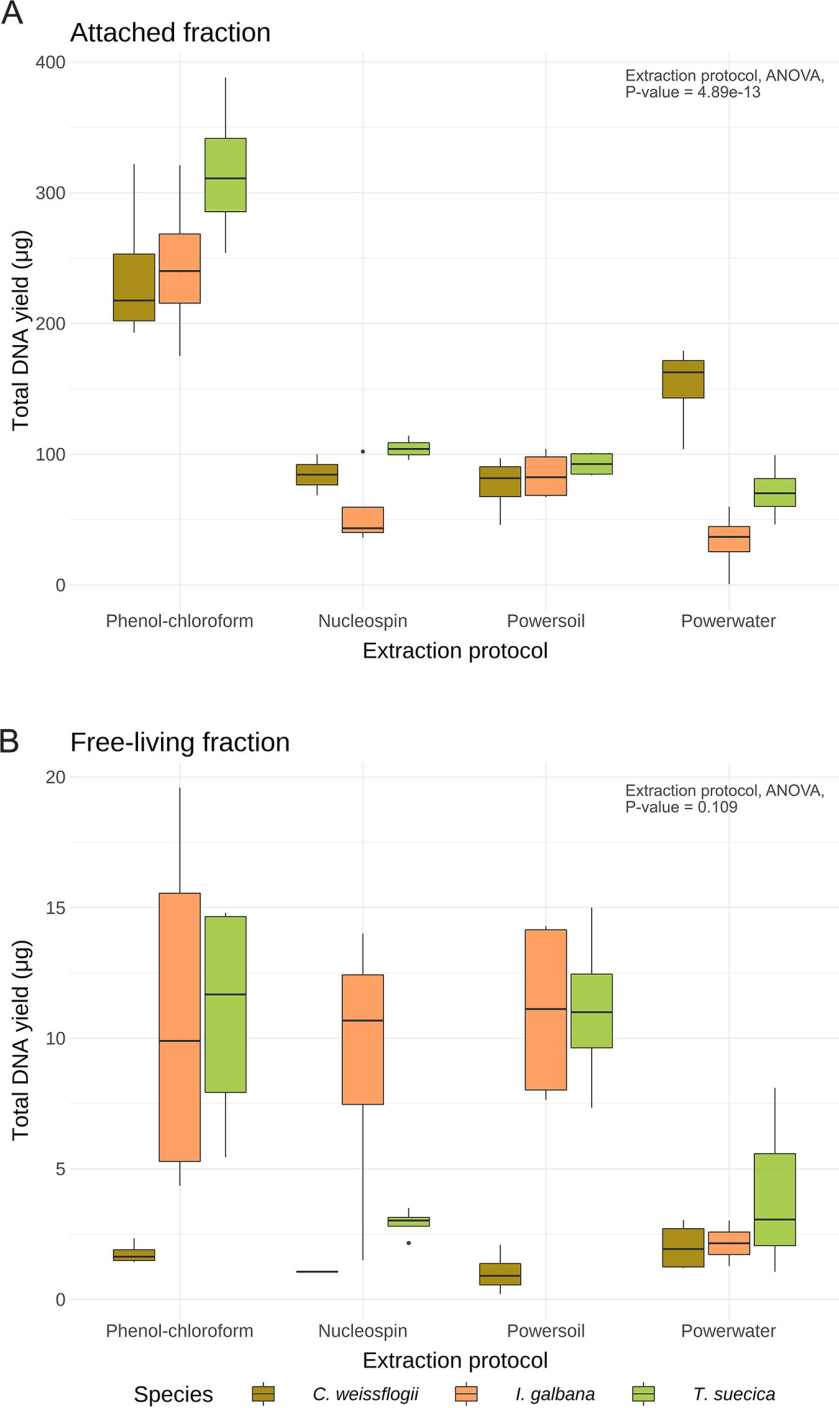
Total DNA yield (μg) from 10-mL xenic microalgal cultures separated, (A) attached, and (B) free-living fractions (*n* = 4). DNA was extracted from three host species of microalgae, Isochrysis galbana (brown), Tetraselmis suecica (orange), and Conticribra weissflogii (green), utilizing four different protocols (Nucleospin, phenol-chloroform, PowerSoil, and PowerWater).

Depending on the DNA extraction method used, the fraction captured, and the algal host species, DNA yield and quality differed greatly (Fig. 1, Table S2). Generally, higher yields and qualities were retrieved from the attached fraction compared to the free-living fraction, with a mean DNA yield of 131.3 μg (range: 0.456 to 388 μg) from filters of the A fraction, whereas filters of the FL fraction had a mean DNA yield of 6.23 μg (range: 0.204 to 19.6 μg), likely due to the lower overall biomass in the FL fraction. In terms of DNA extraction methods, the phenol-chloroform extraction protocol clearly resulted in higher yields and quality for the A fraction compared to all other extraction methods (Tukey’s test after an analysis of variance [ANOVA], adjusted *P* = 0), while the other methods yielded similar amounts and qualities of DNA with small differences across algal host species (Fig. 1A).

For the FL fraction, the phenol-chloroform extraction protocol, PowerSoil kit, and Nucleospin kit yielded similar amounts of DNA, whereas the PowerWater kit yielded smaller amounts across all algal host species (Fig. 1B). According to Tukey’s test after an ANOVA, none of the extraction protocols made a significant difference on the DNA yield for the FL fraction.

The phenol-chloroform extraction protocol has previously been the standard practice in our own and many other laboratories; however, due to its use of the hazardous reagents phenol and chloroform (which may cause carcinogenesis, nephrotoxicity, hepatotoxicity, etc.) and because the protocol is quite time-consuming (5 to 6 h + an over night step (O/N) step), alternatives have been sought out in the form of commercial kits. While the PowerWater kit is optimized for aquatic samples, which should be similar to the ones in the present study, the PowerSoil and Nucleospin kits have also been successfully applied previously to similar samples in our lab. In the samples described here, the phenol-chloroform extraction protocol yielded higher DNA quantity and quality compared to the commercial kits. This is likely because the organic extraction caused less loss of DNA compared to retention of DNA on silica columns, which all three commercial kits rely on. It is possible that the A fraction samples contained more proteins and other contaminants from lysed microalgal and bacterial cells due to the higher total biomass compared to the samples from the FL fraction; hence, these were better separated from the DNA during the phenol-chloroform extraction. However, this should be further corroborated by further studies.

Phenol-chloroform extraction has consistently resulted in high DNA yields in other comparative studies (25, 26, 29), similar to the findings in the present study. However, Douglas et al. (27) obtained low DNA purities utilizing a phenol-chloroform extraction, which is in contrast to the present study and several others, where phenol-chloroform extraction resulted in high DNA quality and quantity (25, 26). Commercial kits such as the Nucleospin, PowerSoil, and PowerWater kits have generally resulted in moderate to small amounts and qualities of DNA in other studies (25–27, 29), but specific kits can also yield similar amounts of DNA compared to phenol-chloroform extraction for specific applications, e.g., Rubin et al. (26) found the Qiagen DNeasy blood and tissue kit to be equally efficient to a phenol-chloroform protocol for DNA extraction from microbial communities associated with ants. Sui et al. (25) also found that high-throughput kits such as the Promega Maxwell HT 96 gDNA Blood Isolation System resulted in DNA quantities comparable to those of phenol-chloroform extraction for different human samples. Although none of the selected commercial kits resulted in DNA quantities or qualities as high as those of phenol-chloroform extraction in the present study, the commercial kits did provide enough DNA for subsequent sequencing with very few exceptions.

### Sequencing data and alpha diversity.

**FIG 2:**
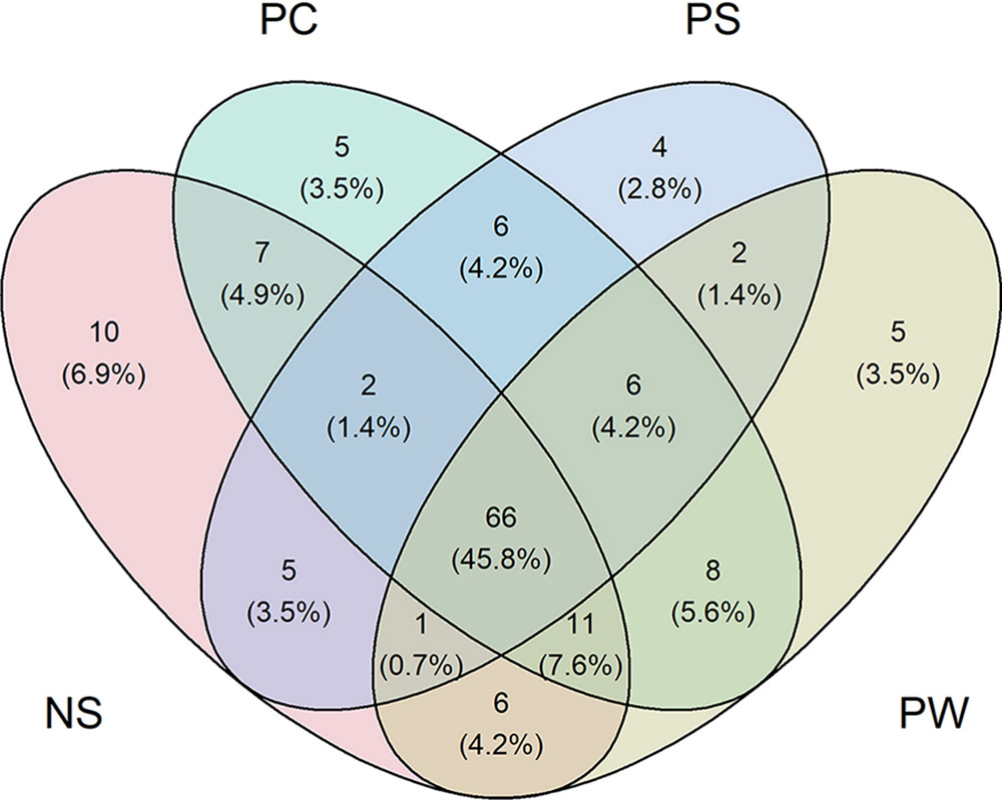
Venn diagram of number and percentages of amplicon sequence variants (ASVs) shared between microalgal microbiomes characterized by 16S amplicon sequencing and DNA extracted with four different protocols; phenol-chloroform (PC, mint), PowerSoil (PS, light blue), PowerWater (PW, lime), and Nucleospin (NS, rose).

**FIG 3:**
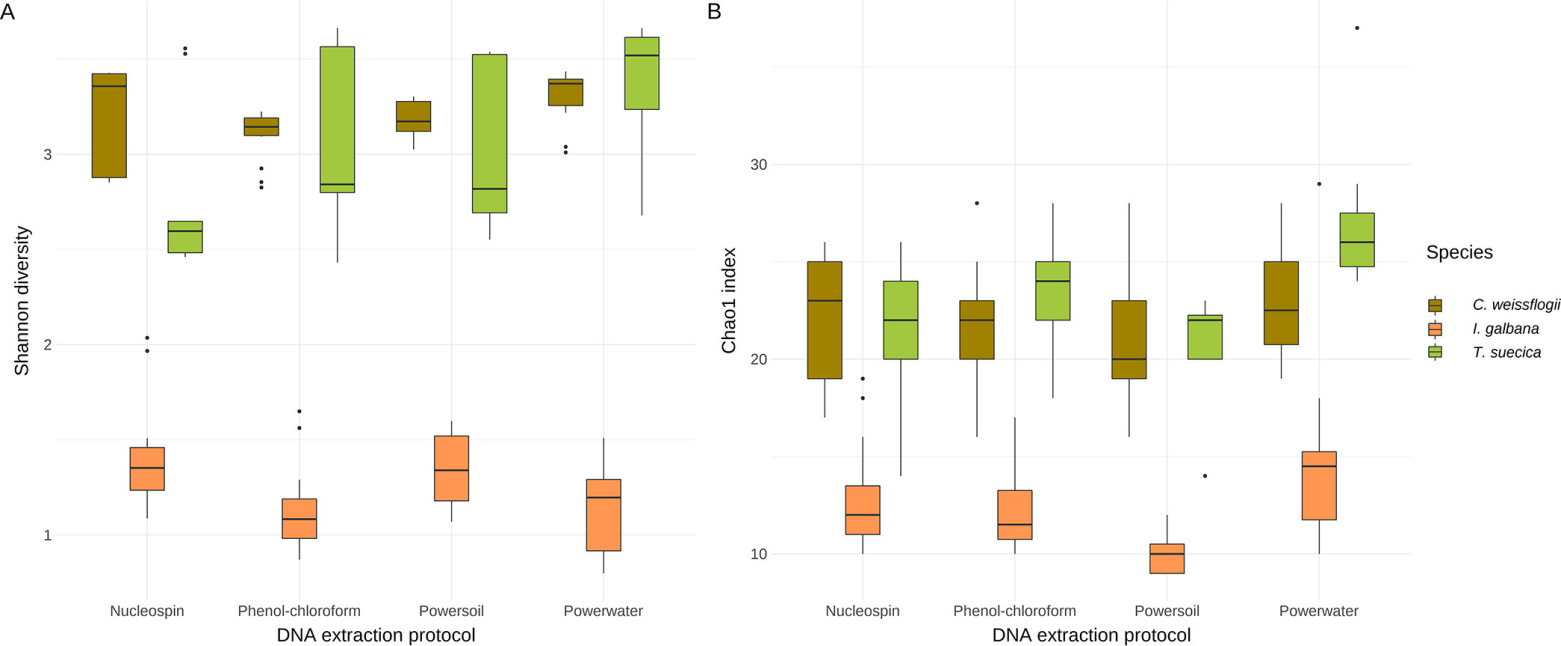
Alpha diversity measures of microbiomes associated with the three host microalgal species *I. galbana* (orange), T. suecica (green), and *C. weissflogii* (brown). (A) Shannon’s diversity index. (B) Chao1 index. DNA was extracted utilizing four different protocols: Nucleospin, phenol-chloroform, PowerSoil, and PowerWater.

**FIG 4:**
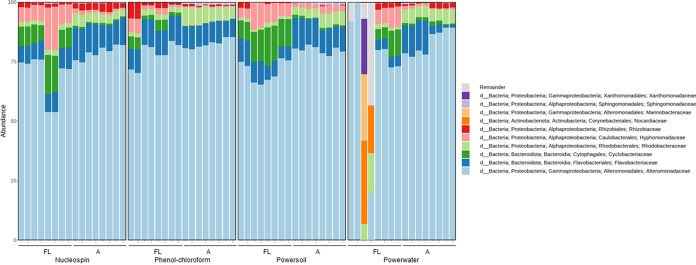
Relative abundance of the top 10 most abundant taxonomic families in microbiome samples associated with *I. galbana*. Samples are grouped by the four DNA extraction protocols utilized and by attached (A) and free-living (FL) fractions. Samples with very low read numbers may show different compositions compared to other replicates.

A total of 43,491,818 paired end reads were obtained from the 16S rRNA gene amplicons, with a mean of 56,818 reads per sample (Table S2). This resulted in a total of 144 amplicon sequence variants (ASVs) after denoising, merging, and filtering out chloroplasts, with a mean ASV frequency per sample of 17,133. In total, 108 of these were detected in samples extracted with the Nucleospin kit, whereas 111, 92, and 105 were detected in samples extracted with the phenol-chloroform, PowerSoil, and PowerWater protocols, respectively (Fig. 2). However, we computed alpha diversity measures (Shannon diversity and Chao1 indices) and compared them across extraction protocols and saw no significant differences (Fig. 3). Shannon diversity and Chao1 index (richness) measures were comparable for microbiomes associated with T. suecica and *C. weissflogii*, but significantly lower in terms of both richness (Chao1) and diversity for microbiomes associated with *I. galbana*, where especially *Alteromonas* dominated the microbiomes (Fig. 4). In general, microbiomes were comprised of 10 to 30 unique ASVs. This points to microbiomes possibly being reduced over time since the microalgae had been in xenic laboratory culture for prolonged periods. Similar species richness and diversity have previously been observed in other amplicon sequencing studies of microalgal microbiomes (22, 24, 30), both from microalgal cultures from culture collections and from microalgae isolated from natural environments. However, other studies have found higher richness in microalgal microbiomes from laboratory cultures (15, 33), demonstrating that factors other than natural or culture conditions and extraction methods affect diversity and species richness in microalgal microbiomes. Alpha rarefaction curves of samples in the present study confirmed that appropriate sequencing depths had been reached (Fig. S2). Together, these findings confirm that relative abundances were suitable for further analyses of microbiome compositions and beta diversity assessments.

### Beta diversity and microbiome compositions.

**FIG 5:**
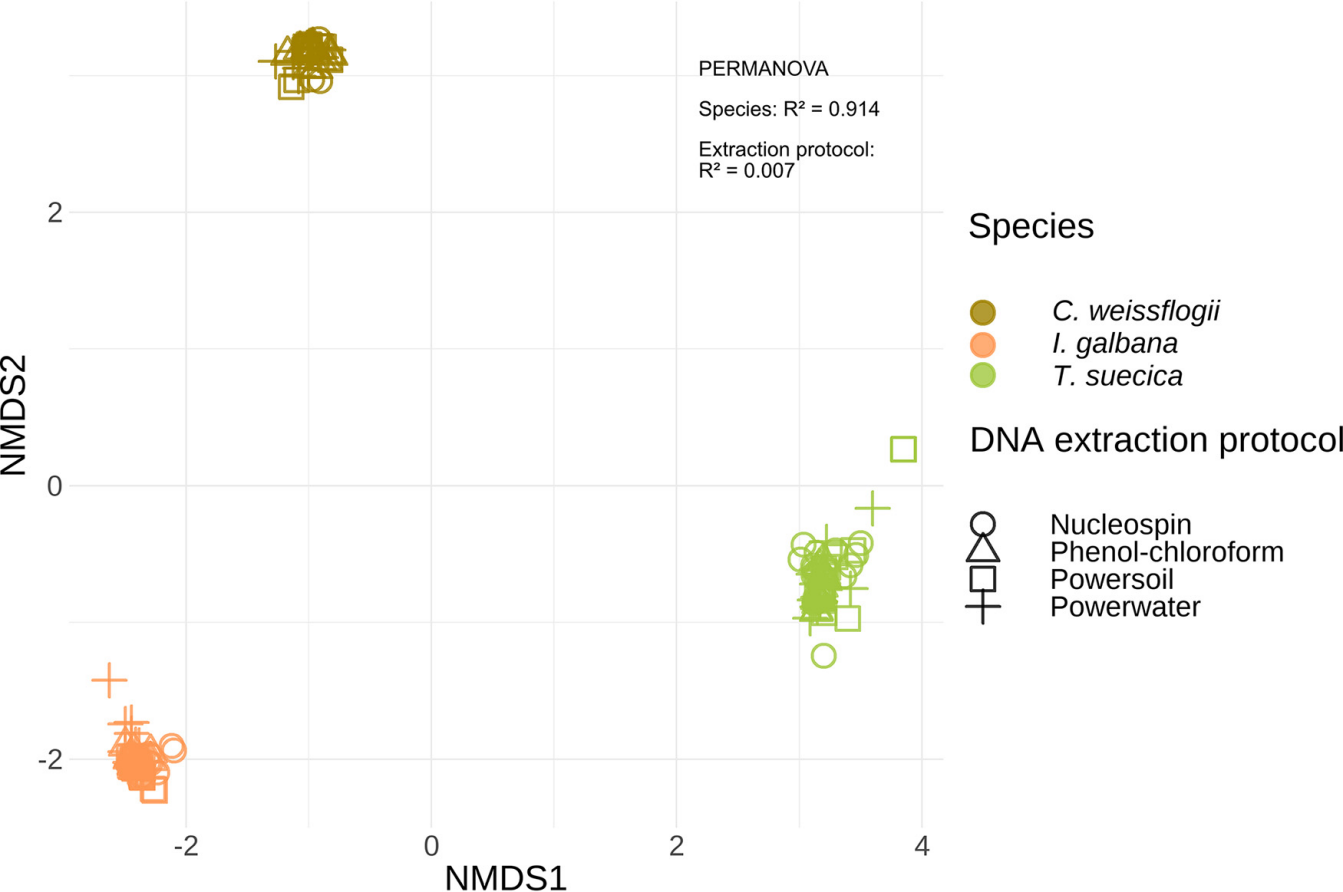
Nonmetric multidimensional scaling (NMDS) plot based on Bray-Curtis dissimilarities of the microbiome compositions of microbiota associated with three species of microalgae: *I. galbana* (brown), T. suecica (orange), and *C. weissflogii* (green). To assess microbiome compositions, DNA was extracted utilizing four different protocols: Nucleospin (circle), phenol-chloroform (triangle), PowerSoil (square), and PowerWater (plus). The majority of beta diversity variation is explained by host species of the microbiome (R^2^ = 0.91, *P* value < 0.001, PERMANOVA, perm. = 999).

Overall, microbiome compositions varied greatly across microalgal host species, and much less within microbiomes from the same host species (Fig. 5). This was confirmed statistically by a permutational multivariate ANOVA (PERMANOVA) (34), as approximately 91% of the variation in the microbiome composition data could be explained by microalgal host species (R^2^ = 0.914, *P* = 0.001, permutations = 999). In contrast, the DNA extraction method had little impact on microbiome composition, representing only 0.7% of the variation (PERMANOVA; R^2^ = 0.007, *P* = 0.001, permutations = 999), which does not reflect the differences in DNA yield and quality seen across the various methods.

Finally, we observed a clear difference in the microbiome composition of each host alga between the A and FL fractions; this was especially clear when we accounted first for host species (Fig. S1) and subsequently for fraction, where approximately 3% of the variation could be explained by this combined effect (PERMANOVA, species:fraction, R^2^ = 0.032, *P* = 0.001, permutations = 999). When we performed a PERMANOVA for the microbiomes associated with each host species individually, fraction was the biggest determinant of variation, explaining around 43% of microbiome composition variation for *I. galbana*-associated microbiomes (PERMANOVA, R^2^ = 0.427, *P* = 0.001, permutations = 999), approximately 64% for T. suecica-associated microbiomes (PERMANOVA, R^2^ = 0.637, *P* = 0.001, permutations = 999), and approximately 56% for *C. weissflogii*-associated microbiomes (PERMANOVA, R^2^ = 0.561, *P* = 0.001, permutations = 999).

These results reflect previous studies of microalgal microbiomes in which host species was a determining factor for microbiome composition in the phycosphere. Although other parameters not evaluated here, such as dissolved oxygen, pH, nitrate, etc., may also drive microbiome composition, other studies have described similar findings. Krohn-Molt et al. (24) found microbiomes associated with *Scenedesmus*, *Micrasterias*, and *Chlorella* to be significantly different across host species as assessed by both 16S rRNA gene amplicon sequencing and metagenomics. Similarly, Ahern et al. (35) found microbiomes associated with isolates of the same diatom species, *Thalassiosira rotula*, to be different between strains, suggesting that microalgal microbiomes may be specific to host species all the way to the strain level; this would be in concordance with the large difference in microbiome composition observed between the distantly related microalgal host species investigated in the present study. Significant differences between attached and free-living fractions of microalgal microbiomes have also been observed in both freshwater (36) and marine species (31). Finally, while some studies have found microbiome composition to be largely impacted by DNA extraction protocols (27, 29), others have found little to no impact, in concordance with this study (25, 26, 28). Samples with especially low biomass can be impacted by contamination from DNA extraction kits and other reagents (37). One study found the PCR master mix to be the primary source of contamination (38), whereas others found nucleic acid extraction kits to be the main sources (39, 40). In the present study, the small but significant effect of DNA extraction kits on microbiome compositions could have been caused by reagent contamination in the DNA extraction kits but could also have been caused by differences in lysis efficiency, especially because the four protocols utilized have different lysis methods. While the bacterial loads in this study were not very high, they were well above the thresholds of 10^3^ bacterial cells/mL recommended by Salter et al. (37); thus, contamination from kits should not greatly impact microbiome composition, which may be the effect seen in this study. These results emphasize the need to assess the impact of extraction methods on microbiome composition for specific sample types, because it seems to be vastly different across extraction methods, bacterial loads, systems, and samples. For the present study, it should be noted that these results are based on 16S rRNA gene amplicon sequencing, which has known limitations such as 16S rRNA gene copy number and primer bias. Hence, other sequencing methods such as shotgun metagenomics could yield different results in terms of the impact of extraction methods, which should be further examined in future studies.

**FIG 6:**
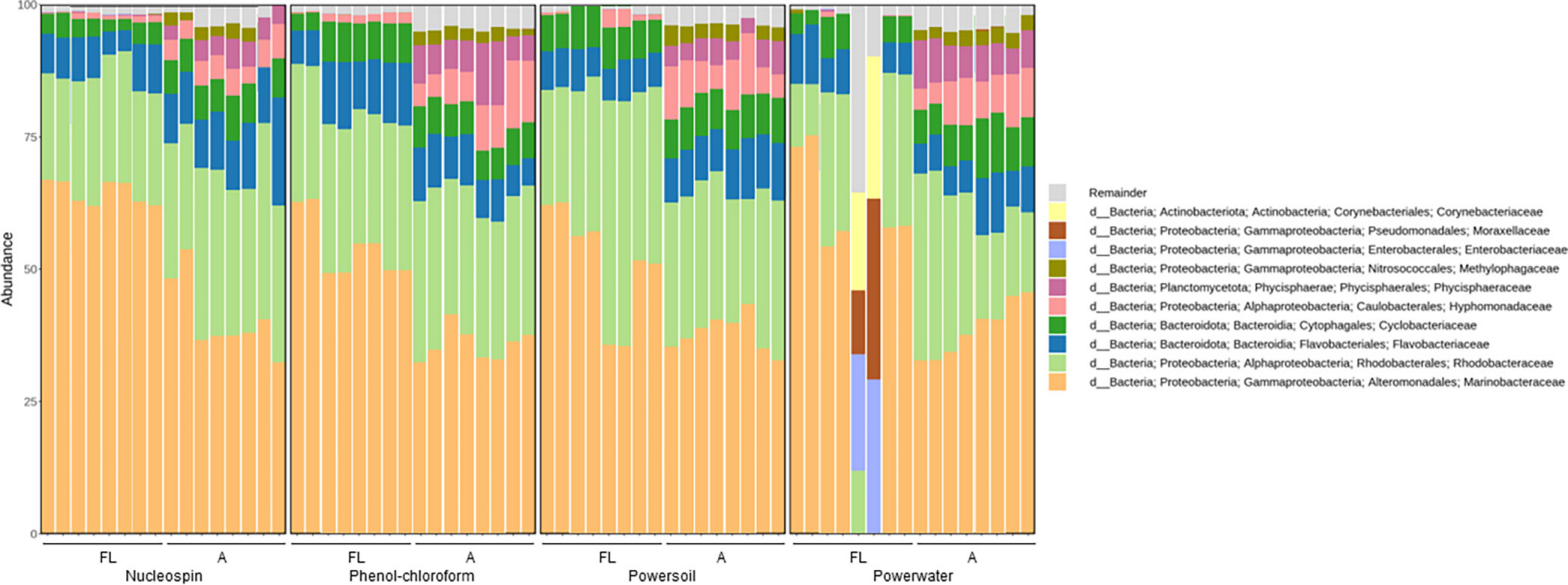
: Relative abundance of the top 10 most abundant taxonomic families in microbiome samples associated with T. suecica. Samples are grouped by the four DNA extraction protocols utilized and by attached (A) and free-living (FL) fractions. Samples with very low read numbers may show different compositions compared to other replicates.

**FIG 7:**
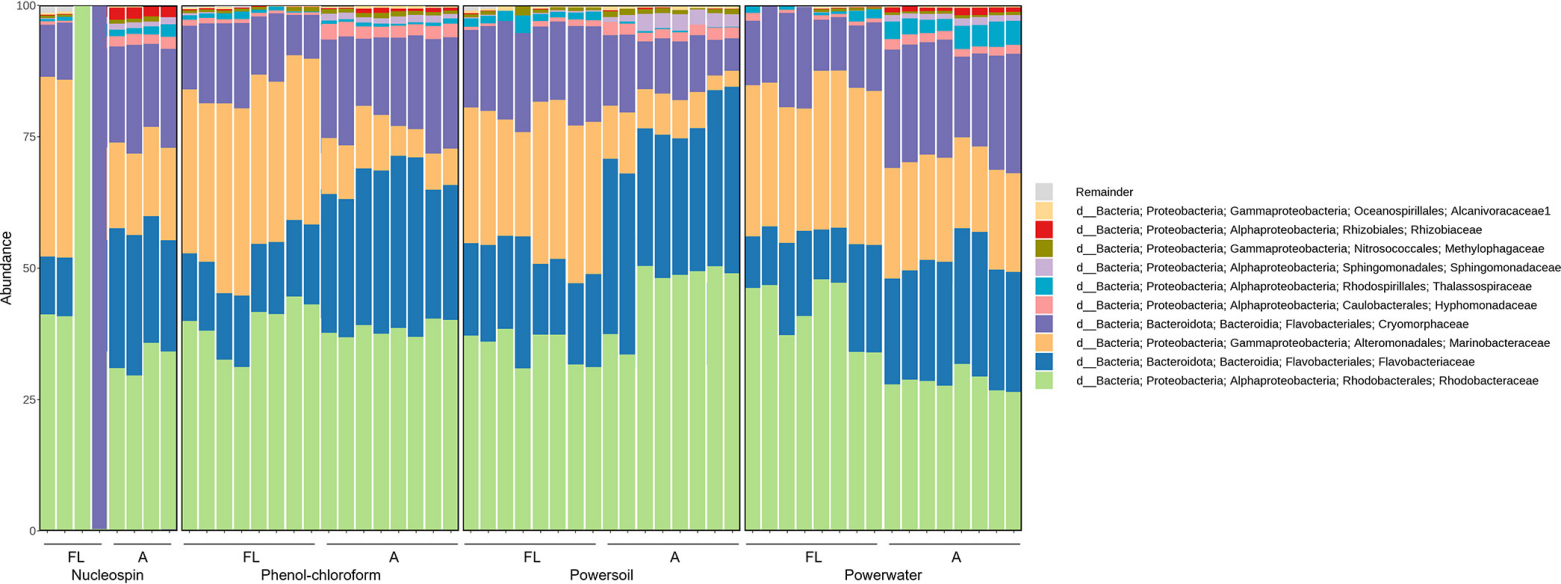
: Relative abundance of the top 10 most abundant taxonomic families in microbiome samples associated with *C. weissflogii*. Samples are grouped by the four DNA extraction protocols utilized and by attached (A) and free-living (FL) fractions. Samples with very low read numbers may show different compositions compared to other replicates.

Overall, the microalgal microbiomes as assessed by 16S rRNA gene amplicon sequencing were dominated by *Proteobacteria*, especially *Gamma*- and *Alphaproteobacteria*, and *Bacteriodetes*, especially the *Flavobacteriaceae* family was abundant in all microbiomes (Fig. 4, 6, and 7). The microbiomes associated with *I. galbana* were dominated largely by *Alteromonas*, whereas the families *Marinobacteraceae* and *Rhodobacteraceae* were prevalent in the T. suecica microbiomes. Finally, the *C. weissflogii* microbiomes were comprised of mainly *Rhodobacteraceae*, *Flavobacteriaceae*, *Marinobacteraceae*, and *Cryomorphaceae*. For the T. suecica microbiome, the A fraction was specifically associated with the families *Methylophagaceae*, *Phycisphaeraceae*, and *Hyphomonadaceae*. Similarly, the A fraction of the *C. weissflogii* microbiomes contained *Rhizobiaceae* in addition to microbiome members prevalent in both the A and FL fractions. *Rhizobiaceae* was also observed at higher abundances in the A fraction than in the FL fraction of the *I. galbana* microbiomes, along with higher abundances of *Rhodobacteraceae* and lower abundances of *Cyclobacteriaceae* and *Hyphomonadaceae*. Engineering the phycosphere microbiome in a beneficial and stable direction requires knowledge of microbial players and may correlate algal health and growth with key actors in the microbiome.

Previous studies of microalgal microbiomes have found equally different compositions across host species to those found in the present study. Some similar patterns of microbiomes being dominated by *Proteobacteria* and *Bacteriodetes* were found previously by Krohn-Molt et al. (24) using 16S rRNA gene amplicon sequencing, although the families within these phyla were mostly different from those seen in this study, with *Sphingomonadaceae* dominating the microbiomes of *Micrasterias* and *Comamonadaceae* dominating the microbiomes of *Scenedesmus*. These families are barely present in the microbiomes characterized in this study, possibly because the microalgal host species in this study are marine while those utilized by Krohn-Molt et al. (24) are freshwater species. In studies with marine algal host species, the microbiomes of diatoms Asterionellopsis glacialis and Nitzschia longissima were dominated by members of the family *Rhodobacteraceae* (22), which was also abundant in the diatom microbiome characterized here. Another abundant member of the *N. longissima* microbiome was an *Oceanicola* sp. (22), belonging to the *Roseobacteraceae* family, which was not observed in our diatom microbiomes. For haptophytes, one study found that the microbiome of *E. huxleyi* was comprised mainly of *Flavobacterales* and *Rhodobacterales* (15), which were also abundant orders in the microbiomes in the present study associated with all three host species. *Alteromonadales* members were also present in the Emiliania huxleyi microbiomes (15), but not nearly as abundant as the *Alteromonadales* members in the microbiome of the closely related *I. galbana*, where more than 75% of the ASVs belonged to the *Alteromonadaceae* family in most samples. In another study characterizing the T. suecica microbiome, Dittmann et al. (16) found the microbiomes to be dominated by the orders *Rhodobacterales* and *Flavobacterales* along with *Phycisphaerales*, *Cytophagales*, and *Caulobacterales*. These orders were all abundant in the T. suecica microbiome characterized in the present study; interestingly enough, *Caulobacterales* and *Phycisphaerales* were highly associated with the A fraction, which was not separated from the FL fraction in the study by Dittmann et al. (16). In the T. suecica microbiome studied here, however, ASVs belonging to the *Marinobacteraceae* family of the order *Alteromonadales* were highly abundant, making up 30% to 60% of the microbiome, and while *Alteromonadales* was among the 10 most abundant orders in the T. suecica microbiome studied by Dittmann et al. (16), it did not comprise more than 5%. This suggests that microalgal microbiomes may have some host selection at the species level, but with variations perhaps to the strain or individual culture level, as suggested by Ahern et al. (35).

ASVs identified as chloroplasts were found mostly in the A fraction, but also in small amounts in some samples from the FL fraction. Samples from the A fraction of microbiomes associated with T. suecica were especially enriched in chloroplast ASVs, with relative abundances of 64% to 95%, whereas the A fraction of *I. galbana* microbiomes only contained 7% to 49% chloroplast ASVs. The A fraction of *C. weissflogii* microbiomes contained very small amounts of chloroplast ASVs, between 0% and 15%, likely due to the difficulty of extracting DNA from the diatom due to its silica shell. Other studies have found that extraction methods which utilize a combination of lysis methods, as opposed to only one at a time as in the present study, resulted in higher diatom DNA yields (41, 42).

### Conclusion.

In conclusion, DNA quality and quantity were greatly impacted by the DNA extraction protocol chosen, especially for the attached fractions of the microalgal microbiomes. A phenol-chloroform extraction protocol resulted in the highest DNA yields and qualities compared to commercial kits. Conversely, DNA extraction protocols had very little impact on microalgal microbiome composition as characterized by 16S rRNA gene amplicon sequencing. Hence, commercial kits for DNA extraction are just as suitable as phenol-chloroform extraction for 16S amplicon-based community composition analyses of microalgal microbiomes, whereas other applications in which high DNA yield and quality is of utmost importance can benefit from utilizing phenol-chloroform extraction. Commercial kits may have other advantages over phenol-chloroform extractions, such as lower reagent toxicity, that can be deemed more important than high DNA quantities in some cases.

Overall, the present study may provide valuable information on extraction method selection for those planning microalgal microbiome studies and underline the need for evaluating the impact of extraction methods for selected systems and samples in microbiome studies.

## MATERIALS AND METHODS

### Cultivation of microalgae.

Three species of xenic microalgae were kindly provided by aquaculture industry partners or purchased from culture collections: Isochrysis galbana (an aquaculture production in Spain), Tetraselmis suecica (an aquaculture production in Greece), and Conticribra weissflogii CCAP 1085/18. *I. galbana* and T. suecica were routinely cultivated in f/2 medium (43, 44) without silica prepared with 3% Instant Ocean (f/2-Si 3% IO; Aquarium Systems). *C. weissflogii* was cultivated in f/2+Si 3% IO.

### Harvest of microbiome and separation of fractions.

*I. galbana*, T. suecica, and *C. weissflogii* were cultivated in 300-mL cultures for 7 days until reaching early stationary phase at 18°C and ~50 μmol m^−2^ s^−1^ with gentle, sterile aeration. Next 10 mL of outgrown cultures was filtered onto 47-mm, Ø (pore size) 5-μm polycarbonate (PC, GE Water & Process Technologies) filters to capture the particle-attached fraction of bacteria in the microbiomes, and the run-through was then sequentially filtered onto 47-mm Ø 0.2-μm PC filters (MontaMil, PCC047020) to capture the free-living fraction of bacteria in the microbiomes. This was done in 16 technical replicates per microalgal species, resulting in a total of 96 filters.

### Enumeration of algal and bacterial cells.

Cell counts of algal and bacterial cells were determined in an experimental setup identical to that of the cultures harvested for microbiome characterization. Xenic cultures of the three microalgae were cultivated for 7 days in 300-mL cultures at 18°C and ~50 μmol m^−2^ s^−1^ with gentle, sterile aeration. Microalgal cell counts were then determined by microscopy counting in a Neubauer-improved chamber, and bacterial cell counts were determined using SYBR Gold and fluorescence microscopy as previously described (45). Five mL of the xenic cultures was filtered onto 25-mm Ø 5-μm PC filters to capture the A fraction of bacteria, and the run-through was then sequentially filtered onto 25-mm Ø 0.2-μm PC filters to capture the FL fraction. Filters were then stained with SYBR Gold and analyzed by fluorescence microscopy (Olympus BX51).

### DNA extractions.

**FIG 8:**
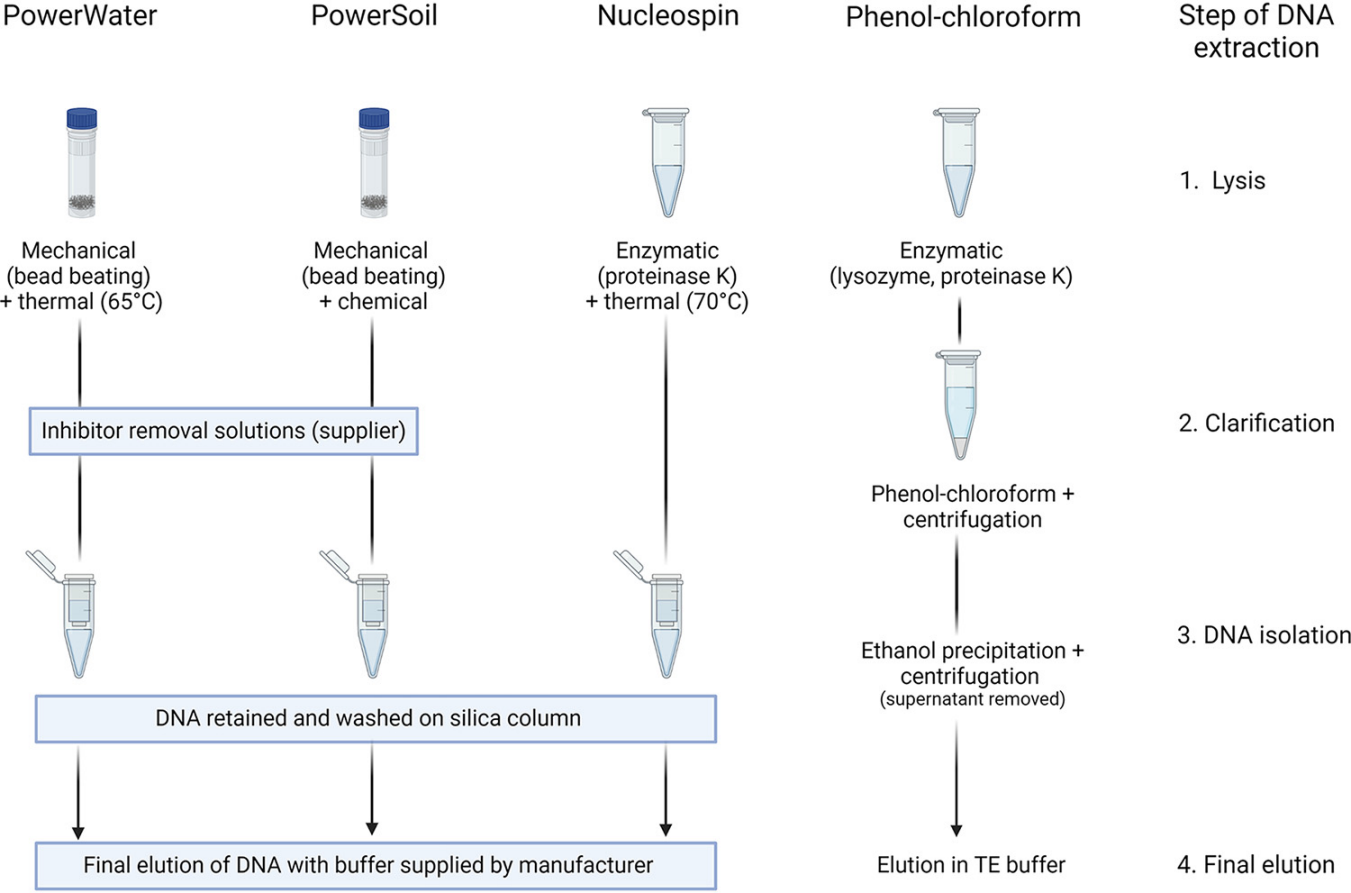
Overview of the main steps of the four DNA extraction protocols with a focus on sample lysis (step 1), clarification of contaminants (step 2), isolation of DNA from samples (step 3), and final elution of DNA (4). Inspired by Vasselon et al. (42).

DNA was extracted from the filters utilizing four different extraction methods or commercial kits. DNA from each microalga and each fraction was extracted in quadruplicate using the DNeasy PowerWater kit (Qiagen), DNeasy PowerSoil kit (Qiagen), NucleoSpin Tissue kit (Macherey-Nagel), and a phenol-chloroform extraction protocol adapted from Boström et al. (46) (Fig. 8). The filters were cut into small pieces using sterile scissors before DNA extraction for all protocols except the DNeasy PowerWater kit, which is optimized for uncut filters. For the commercial kits, the suppliers’ protocols were followed. Both the DNeasy PowerWater kit and the DNeasy PowerSoil kit protocols contain mechanical lysis steps in the form of bead beating, which was applied to the samples. An additional lysis step at 65°C for 10 min was performed as part of the extraction using the DNeasy PowerWater kit as recommended by the supplier for hard-to-lyse samples, whereas the DNeasy PowerSoil kit protocol contains a default thermal lysis step at 70°C for 10 min. The Nucleospin Tissue kit protocol utilizes proteinase K for enzymatic lysis. For the phenol-chloroform protocol, the cut filters were submerged in 1 mL lysis buffer (400 mM NaCl, 750 sucrose, 20 mM EDTA, 50 mM Tris-HCl, 1 mg/mL lysozyme [pH 8.5]) and incubated at 37°C for 30 min. Proteinase K was added to a final concentration of 100 μg/mL along with sodium dodecyl sulfate (SDS) to a final concentration of 1% vol/vol, and samples were then incubated overnight at 55°C with agitation at 60 rpm. Phenol:chloroform:isoamyl alcohol (25:24:1 vol/vol/vol) was added in a volume equal to the combined volume of the added lysis buffer, proteinase K, and SDS. This was mixed by vortexing and subsequently centrifuged for 5 min at 4°C at 20,000 × *g*. Chloroform:isoamylalcohol (24:1 vol/vol) was added at an equal volume to the supernatant. After vortexing and centrifugation for 5 min at 4°C at 20,000 × *g*, the supernatant was transferred and 0.1 volume (compared to supernatant volume) of sodium acetate (3 M) (pH 5.6) was added along with 0.6 volume of ice-cold isopropanol. Samples were then incubated at −20°C for 2 h before centrifugation for 20 min at 4°C at 20,000 × *g*. The supernatant was then discarded and the pellet washed with 500 μL ice-cold ethanol (70% vol/vol) and centrifuged for 20 min at 4°C at 20,000 × *g*. Finally, the supernatant was discarded, and the air-dried pellet was resuspended in 50 μL TE buffer pre-warmed to 56°C. DNA concentrations were measured using a Qubit 2.0 Fluorometer with the high sensitivity (HS) assay kit, and DNA quality and purity were determined using a DeNovix DS-11+ spectrophotometer. Quality assessments were made by assigning pluses based on the 260/280 and 260/230 ratios and spectral patterns, which indicated purity.

### Amplicon sequencing of 16S rRNA gene.

The V3-V4 region of the 16S rRNA gene of all samples was amplified by PCR using the primer set 341f (5′-CCTACGGGNGGCWGCAG-3′) and 805r (5′-GACTACHVGGGTATCTAATCC-3′) (47) with 30 different barcodes (Table S1). Amplification was performed using TEMPase Hot Start 2× Master Mix (Ampliqon), 10 μM forward primer, and 10 μM reverse primer. The PCR program consisted of an initial denaturation step of 15 min at 95°C; 30 cycles of denaturation for 30 s at 95°C, annealing for 30 s at 60°C, and elongation for 30 s at 72°C; and finally an elongation step at 72°C for 5 min. PCR products were pooled in equal amounts according to the barcodes as determined by measurement with the HS assay kit and a Qubit 2.0 Fluorometer and sequenced on an Illumina NovaSeq 6000 instrument with paired-end 250-bp reads (Novogene). To obtain enough reads per sample, the amplicons were sequenced in several lanes on an Illumina Flow Cell, resulting in two additional technical replicates per sample.

### Analysis of sequencing data.

Sequencing data were analyzed using QIIME 2 version 2021.8 (48) and R version 4.1.2. The multiplexed raw reads with barcodes in-sequence were imported into QIIME 2 and de-multiplexed using the cutadapt demux-paired plugin (49). The demultiplexed data were denoised and dereplicated, and the reads were merged using the DADA2 plugin (50). An ASV table was also constructed using the DADA2 plugin of QIIME 2 (50). The taxonomy of reads was assigned by the feature-classifier plugin (51, 52) based on a classifier trained on the Silva database 138 (53) with the primers used for PCR amplification mentioned above and 99% similarity OTUs. Then, all reads classified as chloroplasts as well as reads present in less than two samples were filtered out before further analysis with the q2-taxa filter plugin (48). For phylogenetic analysis, a phylogenetic tree was created using MAFFT and FastTree (54, 55), and phylogenetic analyses of alpha and beta diversity were performed using the diversity plugin (56) at an even sampling depth of 3,000. Significant differences in beta diversity were tested based on Bray-Curtis dissimilarities by PERMANOVA with the beta-group-significance and adonis plugins (34) on the whole data set as well as subsets by host algal species. Based on the Bray-Curtis dissimilarity matrix, which was imported into R v4.1.2 with the qiime2R package (57), nonmetric multidimensional scaling (NMDS) was performed using the metaMDS function of the vegan package (58).

### Data availability.

The raw sequencing data are available at the NCBI SRA under BioProject ID PRJNA855439, Biosample numbers SAMN29492049, SAMN29492050, SAMN29492051, SAMN29492052, SAMN29492053, SAMN29492054, SAMN29492055, and SAMN29492056.
