# Supplementary material for: Sequence-Based Characterization of Microalgal Microbiomes: Impact of DNA Extraction Protocol on Yield and Community Composition
Source: Microbiol Spectr. 2023 Mar 28;11(2):e03408-22. doi: 10.1128/spectrum.03408-22 (PMC10100885; doi:10.1128/spectrum.03408-22)
Supplement: Supplemental file 1 — Supplemental material. Download spectrum.03408-22-s0001.pdf, PDF file, 2.6 MB [file spectrum.03408-22-s0001.pdf]

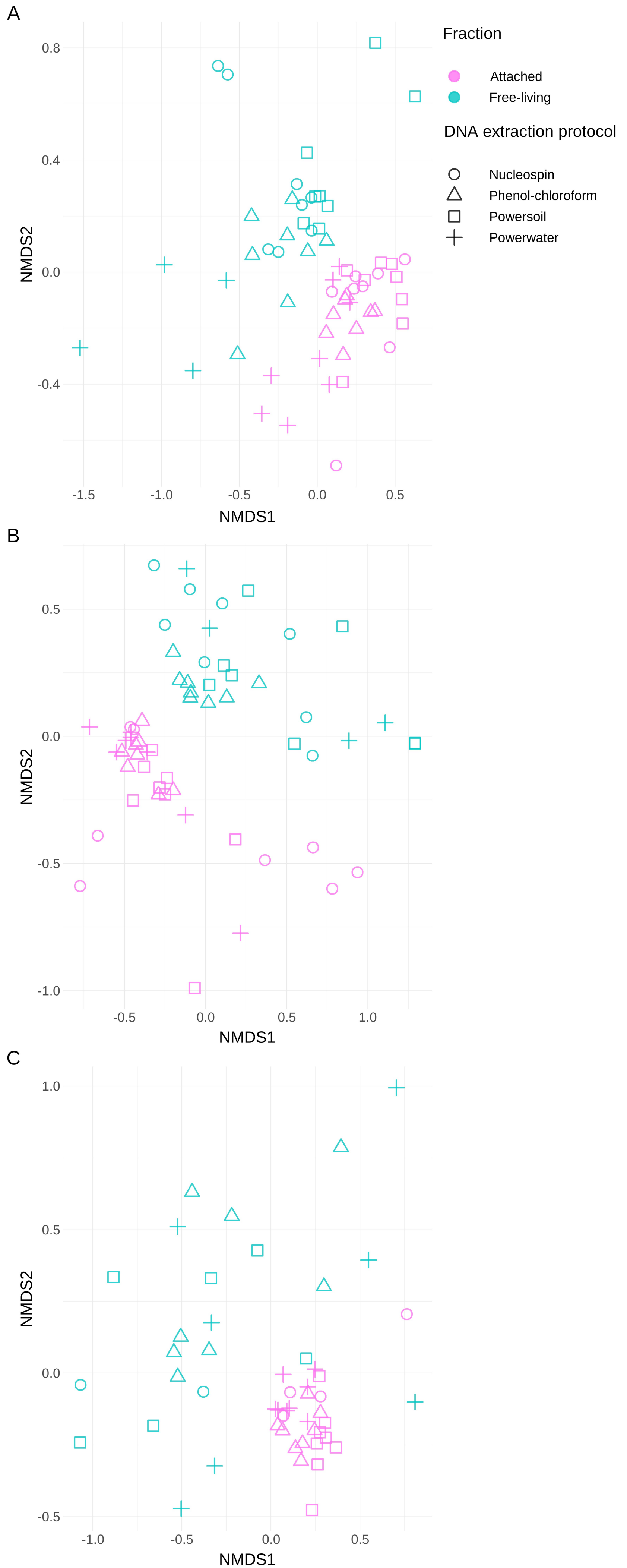

A
B
C
NMDS1
NMDS2
Fraction
Attached
Free-living
DNA extraction protocol
Nucleospin
Phenol-chloroform
Powersoil
Powerwater

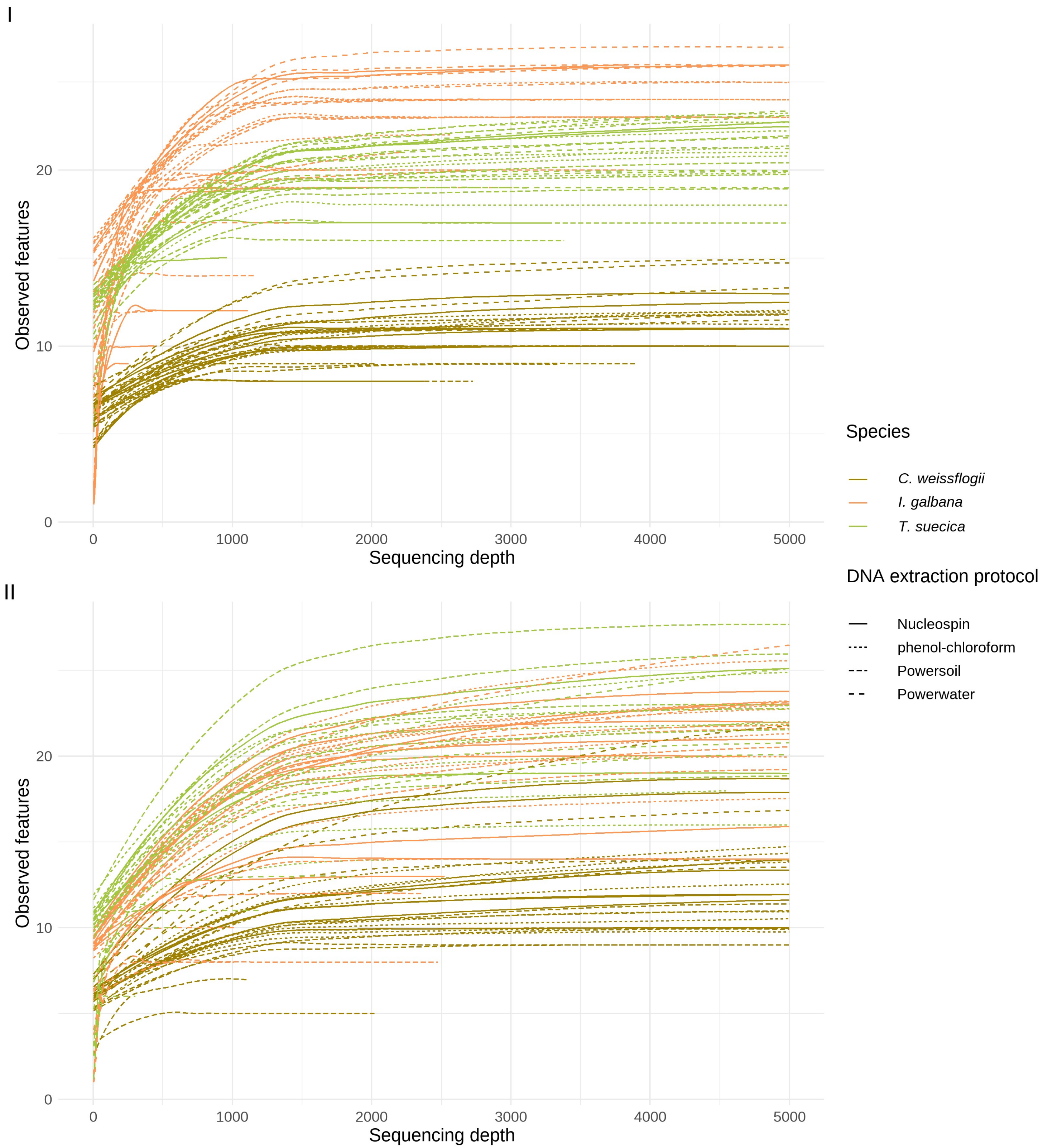
I
II
Observed features
Sequencing depth
0
10
20
0
1000
2000
3000
4000
5000
Species
C. weissflogii
I. galbana
T. suecica
DNA extraction protocol
Nucleospin
phenol-chloroform
Powersoil
Powerwater

Supplementary figure legends

Figure S1: NMDS plots based on Bray Curtis dissimilarities separated by host microalgal species. **A;** *I. galbana*-associated microbiomes, **B;** *T. suecica*-associated microbiomes, and **C;** *C. weissflogii*-associated microbiomes. Microbiomes were separated in attached (A, pink) and free-living (FL, blue) fraction, and DNA extracted utilizing four different protocols, Nucleospin (circle), phenol-chloroform (trangle), Powersoil (square), and Powerwater (plus). When accounting for host species, part of the variation between microbiome compositions are explained by the fraction (species:fraction, $R^2 = 0.032$, p-value < 0.001, PERMANOVA, perm. = 999).

Figure S2: Alpha rarefaction curves based on observed features in samples from the I; attached and II; free-living fraction of the microbiomes associated with *Isochrysis galbana* (orange), *Conticribra weissflogii* (brown), and *Tetraselmis suecica* (green). Microbiome DNA was extracted utilizing Nucleospin (solid line), phenol-chloroform (dotted line), Powersoil (small dashed line), and Powerwater (larger dashed line) extraction protocols. Max sequencing depth was specified at 5,000 with steps of 100.

Table S1: List of barcodes and primers utilized for amplification of the V3-V4 region of the 16S rRNA gene in DNA from the microalgal microbiomes of *I. galbana, T. suecica,* and *C. weissflogii*. Primers and barcodes from Klindworth et al., 2013.

Barcode no.	Barcode	Forward primer (with barcode)	Reverse primer (with barcode)
1	TTTTAATC	TTTTAATCCCTACGGGNGGCWGCAG	TTTTAATCGACTACHVGGGTATCTAATCC
2	ATAATTAG	ATAATTAGCCTACGGGNGGCWGCAG	ATAATTAGGACTACHVGGGTATCTAATCC
3	ACCAAATT	ACCAAATTCCTACGGGNGGCWGCAG	ACCAAATTGACTACHVGGGTATCTAATCC
4	CTTATCAA	CTTATCAACCTACGGGNGGCWGCAG	CTTATCAAGACTACHVGGGTATCTAATCC
5	TGATCATT	TGATCATTCCTACGGGNGGCWGCAG	TGATCATTGACTACHVGGGTATCTAATCC
6	AGAATCTA	AGAATCTACCTACGGGNGGCWGCAG	AGAATCTAGACTACHVGGGTATCTAATCC
7	TCAAGAAA	TCAAGAAACCTACGGGNGGCWGCAG	TCAAGAAAGACTACHVGGGTATCTAATCC
8	ATCGAAAT	ATCGAAATCCTACGGGNGGCWGCAG	ATCGAAATGACTACHVGGGTATCTAATCC
9	ACATTTAC	ACATTTACCCTACGGGNGGCWGCAG	ACATTTACGACTACHVGGGTATCTAATCC
10	TAGAAAAC	TAGAAAACCCTACGGGNGGCWGCAG	TAGAAAACGACTACHVGGGTATCTAATCC
11	TTATCACC	TTATCACCCCTACGGGNGGCWGCAG	TTATCACCGACTACHVGGGTATCTAATCC
12	AATAGGGT	AATAGGGTCCTACGGGNGGCWGCAG	AATAGGGTGACTACHVGGGTATCTAATCC
13	ATTGCTGA	ATTGCTGACCTACGGGNGGCWGCAG	ATTGCTGAGACTACHVGGGTATCTAATCC
14	TGAGTTCT	TGAGTTCTCCTACGGGNGGCWGCAG	TGAGTTCTGACTACHVGGGTATCTAATCC
15	GGCTATTT	GGCTATTTCCTACGGGNGGCWGCAG	GGCTATTTGACTACHVGGGTATCTAATCC
16	CAAGAGAT	CAAGAGATCCTACGGGNGGCWGCAG	CAAGAGATGACTACHVGGGTATCTAATCC
17	GGAATACA	GGAATACACCTACGGGNGGCWGCAG	GGAATACAGACTACHVGGGTATCTAATCC
18	AAGGCAAT	AAGGCAATCCTACGGGNGGCWGCAG	AAGGCAATGACTACHVGGGTATCTAATCC
19	ACAAAACG	ACAAAACGCCTACGGGNGGCWGCAG	ACAAAACGGACTACHVGGGTATCTAATCC
21	TTGAGTGA	TTGAGTGACCTACGGGNGGCWGCAG	TTGAGTGAGACTACHVGGGTATCTAATCC
22	GCTTCTGA	GCTTCTGACCTACGGGNGGCWGCAG	GCTTCTGAGACTACHVGGGTATCTAATCC
23	GGCAAGAT	GGCAAGATCCTACGGGNGGCWGCAG	GGCAAGATGACTACHVGGGTATCTAATCC
24	GTGCTTTC	GTGCTTTCCCTACGGGNGGCWGCAG	GTGCTTTCGACTACHVGGGTATCTAATCC
25	ACACACTG	ACACACTGCCTACGGGNGGCWGCAG	ACACACTGGACTACHVGGGTATCTAATCC
26	CGATTCTG	CGATTCTGCCTACGGGNGGCWGCAG	CGATTCTGGACTACHVGGGTATCTAATCC
27	GCAGAGTT	GCAGAGTTCCTACGGGNGGCWGCAG	GCAGAGTTGACTACHVGGGTATCTAATCC
28	CGTCCTAT	CGTCCTATCCTACGGGNGGCWGCAG	CGTCCTATGACTACHVGGGTATCTAATCC
30	GCTTGGTT	GCTTGGTTCCTACGGGNGGCWGCAG	GCTTGGTTGACTACHVGGGTATCTAATCC
31	ACAGGCTT	ACAGGCTTCCTACGGGNGGCWGCAG	ACAGGCTTGACTACHVGGGTATCTAATCC
33	TGACGCTT	TGACGCTTCCTACGGGNGGCWGCAG	TGACGCTTGACTACHVGGGTATCTAATCC

Table S2: Sample DNA quantity and quality and resulting sequencing reads of DNA extracted from 10 ml culture of microbiomes associated with the three microalgal host species *Isochrysis galbana, Tetraselmis suecica,* and *Conticribra weissflogii* with the four DNA extraction protocols Nucleospin, Powerwater, Powersoil, and phenol-chloroform, and separated into the attached (A) fraction and free-living (FL) fraction by filtration.

**Sample**	**Species**	**DNA extraction protocol**	**Fraction**	**DNA yield (µg)**	**DNA quality**	**Sequencing reads**
NS-I15	*Isochrysis galbana*	Nucleospin	A	41.4	+	32,955
NS-I12	*Isochrysis galbana*	Nucleospin	FL	14.0	+	87,052
NS-I25	*Isochrysis galbana*	Nucleospin	A	45.4	++	79,673
NS-I22	*Isochrysis galbana*	Nucleospin	FL	11.9	+	21,221
NS-I35	*Isochrysis galbana*	Nucleospin	A	36.2	+	34,110
NS-I32	*Isochrysis galbana*	Nucleospin	FL	1.50	+	53,200
NS-I45	*Isochrysis galbana*	Nucleospin	A	102	+	15,318
NS-I42	*Isochrysis galbana*	Nucleospin	FL	9.46	+	36,357
PC-I15	*Isochrysis galbana*	Phenol-chloroform	A	175	+++	59,322
PC-I12	*Isochrysis galbana*	Phenol-chloroform	FL	4.35	++	91,466
PC-I25	*Isochrysis galbana*	Phenol-chloroform	A	229	+++	14,642
PC-I22	*Isochrysis galbana*	Phenol-chloroform	FL	14.2	++	30,772
PC-I35	*Isochrysis galbana*	Phenol-chloroform	A	251	+++	241,979
PC-I32	*Isochrysis galbana*	Phenol-chloroform	FL	5.60	+	31,392
PC-I45	*Isochrysis galbana*	Phenol-chloroform	A	321	++	114,009
PC-I42	*Isochrysis galbana*	Phenol-chloroform	FL	19.6	++	85,339
PS-I15	*Isochrysis galbana*	Powersoil	A	104	+	12,250
PS-I12	*Isochrysis galbana*	Powersoil	FL	14.3	+	30,865
PS-I25	*Isochrysis galbana*	Powersoil	A	95.8	+	13,932
PS-I22	*Isochrysis galbana*	Powersoil	FL	7.64	+	3,795
PS-I35	*Isochrysis galbana*	Powersoil	A	69.0	+	26,545
PS-I32	*Isochrysis galbana*	Powersoil	FL	8.14	+	15,586
PS-I45	*Isochrysis galbana*	Powersoil	A	67.0	++	10,341
PS-I42	*Isochrysis galbana*	Powersoil	FL	14.1	+	42,366
PW-I15	*Isochrysis galbana*	Powerwater	A	0.456	+	35,357
PW-I12	*Isochrysis galbana*	Powerwater	FL	NA	NA	173
PW-I25	*Isochrysis galbana*	Powerwater	A	39.6	++	48,014
PW-I22	*Isochrysis galbana*	Powerwater	FL	NA	NA	208
PW-I35	*Isochrysis galbana*	Powerwater	A	59.8	+	18,496
PW-I32	*Isochrysis galbana*	Powerwater	FL	1.28	+	40,375
PW-I45	*Isochrysis galbana*	Powerwater	A	33.8	+	44,577
PW-I42	*Isochrysis galbana*	Powerwater	FL	3.02	+	66,792
NS-TS15	*Tetraselmis suecica*	Nucleospin	A	114	++	12,068
NS-TS12	*Tetraselmis suecica*	Nucleospin	FL	2.16	+	29,460
NS-TS25	*Tetraselmis suecica*	Nucleospin	A	95.6	++	71,007

NS-TS22	*Tetraselmis suecica*	Nucleospin	FL	3.50	+	10,974
NS-TS35	*Tetraselmis suecica*	Nucleospin	A	107	+	47,167
NS-TS32	*Tetraselmis suecica*	Nucleospin	FL	3.02	+	20,123
NS-TS45	*Tetraselmis suecica*	Nucleospin	A	101	+++	3,374
NS-TS42	*Tetraselmis suecica*	Nucleospin	FL	3.02	++	23,511
PC-TS15	*Tetraselmis suecica*	Phenol-chloroform	A	326	+++	70,725
PC-TS12	*Tetraselmis suecica*	Phenol-chloroform	FL	5.45	+	35,672
PC-TS25	*Tetraselmis suecica*	Phenol-chloroform	A	388	++	31,862
PC-TS22	*Tetraselmis suecica*	Phenol-chloroform	FL	14.8	++	52,389
PC-TS35	*Tetraselmis suecica*	Phenol-chloroform	A	296	++	80,419
PC-TS32	*Tetraselmis suecica*	Phenol-chloroform	FL	8.75	+	46,347
PC-TS45	*Tetraselmis suecica*	Phenol-chloroform	A	254	+++	77,745
PC-TS42	*Tetraselmis suecica*	Phenol-chloroform	FL	14.6	++	132,471
PS-TS15	*Tetraselmis suecica*	Powersoil	A	85.0	+	54,452
PS-TS12	*Tetraselmis suecica*	Powersoil	FL	11.6	+	51,265
PS-TS25	*Tetraselmis suecica*	Powersoil	A	83.8	+	39,950
PS-TS22	*Tetraselmis suecica*	Powersoil	FL	10.4	+	4,456
PS-TS35	*Tetraselmis suecica*	Powersoil	A	100	++	49,222
PS-TS32	*Tetraselmis suecica*	Powersoil	FL	15.0	+	10,168
PS-TS45	*Tetraselmis suecica*	Powersoil	A	101	+	26,310
PS-TS42	*Tetraselmis suecica*	Powersoil	FL	7.34	+	40,365
PW-TS15	*Tetraselmis suecica*	Powerwater	A	75.4	++	107,230
PW-TS12	*Tetraselmis suecica*	Powerwater	FL	1.06	+	1,292
PW-TS25	*Tetraselmis suecica*	Powerwater	A	64.6	+	144,338
PW-TS22	*Tetraselmis suecica*	Powerwater	FL	3.06	+	5,495
PW-TS35	*Tetraselmis suecica*	Powerwater	A	99.2	++	61,812
PW-TS32	*Tetraselmis suecica*	Powerwater	FL	NA	NA	436
PW-TS45	*Tetraselmis suecica*	Powerwater	A	46.2	+	48,167
PW-TS42	*Tetraselmis suecica*	Powerwater	FL	8.10	+	439,404
NS-TW15	*Conticribra weissflogii*	Nucleospin	A	100	++	5,571
NS-TW12	*Conticribra weissflogii*	Nucleospin	FL	1.06	+	38,330
NS-TW25	*Conticribra weissflogii*	Nucleospin	A	68.6	++	145,811
NS-TW22	*Conticribra weissflogii*	Nucleospin	FL	NA	NA	50
NS-TW35	*Conticribra weissflogii*	Nucleospin	A	NA	NA	0
NS-TW32	*Conticribra weissflogii*	Nucleospin	FL	NA	NA	0
NS-TW45	*Conticribra weissflogii*	Nucleospin	A	NA	NA	1
NS-TW42	*Conticribra weissflogii*	Nucleospin	FL	NA	NA	58
PC-TW15	*Conticribra weissflogii*	Phenol-chloroform	A	322	+++	38,746
PC-TW12	*Conticribra weissflogii*	Phenol-chloroform	FL	2.34	+	27,139
PC-TW25	*Conticribra weissflogii*	Phenol-chloroform	A	230	+++	43,446
PC-TW22	*Conticribra weissflogii*	Phenol-chloroform	FL	1.51	++	25,486

PC-TW35	*Conticribra weissflogii*	Phenol-chloroform	A	205	++	141,386
PC-TW32	*Conticribra weissflogii*	Phenol-chloroform	FL	1.76	+	14,342
PC-TW45	*Conticribra weissflogii*	Phenol-chloroform	A	193	+++	28,148
PC-TW42	*Conticribra weissflogii*	Phenol-chloroform	FL	1.43	++	79,205
PS-TW15	*Conticribra weissflogii*	Powersoil	A	46.0	+	10,148
PS-TW12	*Conticribra weissflogii*	Powersoil	FL	1.13	+	36,638
PS-TW25	*Conticribra weissflogii*	Powersoil	A	96.8	+	16,983
PS-TW22	*Conticribra weissflogii*	Powersoil	FL	2.09	+	1,206
PS-TW35	*Conticribra weissflogii*	Powersoil	A	88.4	+	17,898
PS-TW32	*Conticribra weissflogii*	Powersoil	FL	0.676	+	60,955
PS-TW45	*Conticribra weissflogii*	Powersoil	A	74.8	+	50,373
PS-TW42	*Conticribra weissflogii*	Powersoil	FL	0.204	+	23,345
PW-TW15	*Conticribra weissflogii*	Powerwater	A	179	++	35,480
PW-TW12	*Conticribra weissflogii*	Powerwater	FL	1.26	+	2,322
PW-TW25	*Conticribra weissflogii*	Powerwater	A	169	+	241,900
PW-TW22	*Conticribra weissflogii*	Powerwater	FL	1.20	+	3,544
PW-TW35	*Conticribra weissflogii*	Powerwater	A	105	++	102,733
PW-TW32	*Conticribra weissflogii*	Powerwater	FL	2.60	+	53,141
PW-TW45	*Conticribra weissflogii*	Powerwater	A	156	++	865,612
PW-TW42	*Conticribra weissflogii*	Powerwater	FL	3.04	+	76,392
